# Family Sense of Coherence Scale: A Confirmatory Factor Analysis in a Portuguese Sample

**DOI:** 10.3389/fpsyg.2021.762357

**Published:** 2022-01-13

**Authors:** Francis Anne T. Carneiro, Vanessa F. Salvador, Pedro A. Costa, Isabel P. Leal

**Affiliations:** ^1^William James Center for Research, Lisbon, Portugal; ^2^Applied Psychology Research Center – Capabilities and Inclusion, ISPA – University Institute, Lisbon, Portugal

**Keywords:** factorial analysis, family sense of coherence scale, Portuguese caregivers, psychometric properties, resilience, salutogenic model

## Abstract

**Background:** Family sense of coherence (FSOC) can be defined as the cognitive map of a family that enables the family to deal with stress during their lifetime. FSOC is the degree to which a family perceives family life as comprehensible, manageable, and meaningful. To the best of our knowledge, few studies have used this scale, and very few have evaluated FSOC Scale psychometric properties.

**Objective:** This study aimed to evaluate the psychometric properties of the original FSOC Scale in a sample of Portuguese caregivers of children aged between 10 and 15 years.

**Methods:** A total of 329 caregivers completed a sociodemographic questionnaire and the FSOC Scale. Analyses were performed to evaluate the factor structure of the FSOC Scale with 26 items as well as composite reliability, internal consistency, convergent-related validity, and discriminant-related validity of the scale scores.

**Results:** The findings supported a three-factor solution for a 13-item version that maintains the original FSOC Scale structure. The three FSOC dimensions presented a good fit to the data. Cronbach’s alpha, composite reliability, and convergent-related validity were considered very good for the FSOC Scale (α = 0.956; CR = 0.974; AVE = 0.689). No evidence of discriminant-related validity was found for the dimensions of FSOC.

**Conclusion:** The findings support the use of the Portuguese FSOC Scale for research and clinical purposes with Portuguese caregivers. Future research is necessary to further develop a European Portuguese version of the FSOC Scale.

**Implications:** This study provides a psychometric evaluation of FSOC Scale characteristics in a Portuguese sample. The results are helpful for clinicians and family therapists who work with families since it could help them to assess the resources of families and their ability to cope with adversity and enhance their strengths.

## Introduction

Family sense of coherence (FSOC) is a concept developed from the theoretical framework of Antonovsky. According to the salutogenic model of the mentioned author, health is perceived as a continuum from health (*ease*) to disease (*dis-ease*), rather than a “health vs. disease” dichotomy as postulated by the traditional pathogenic model (Antonovsky, 1979, 1987b). It postulates that stressors are inherent to the life of an individual, thus *heterostasis* is considered normative. This means that “The normal state of the human organism is one of disorder and conflict rather than of stability and homeostasis” (Sagy and Antonovsky, 2000, p. 156). Stress *per se* is not considered pathological, and it is a normative and essential stimulus that can introduce factors that could promote healthy change (Antonovsky, 1996). Then, it is important to explore the origins of a successful and healthy coping with stress, instead of focusing merely on the disease etiology. The salutogenic model emphasizes individual strengths and abilities to achieve a successful adjustment and focuses on why some individuals seem to preserve health and wellbeing and are able to successfully cope with daily life stressors and tensions (Antonovsky and Sagy, 1986). The model has two key components: sense of coherence (SOC) and generalized resistance resources (GRR). GRR includes material, biological, social, and psychosocial factors (e.g., intelligence, financial legacy, self-esteem, healthy behaviors, social support, life perspectives, and others) that enables the life perspective of an individual to be seen as consistent, structured, and comprehensive (Antonovsky and Sourani, 1988; Antonovsky, 1993; Söderhamn and Holmgren, 2004; Lindström and Eriksson, 2006). The ability to use these resources is promoted by SOC which could be defined as the ability of an individual to maintain orientation, organization, and structure, regardless of life events and the severity of an individual (Antonovsky, 1979). SOC is a construct that includes the development of coping strategies, rather than a coping style, and can be considered as a global view that things will work out as well as it can be expected (Antonovsky, 1987a). Vast literature highlights that an increased SOC is significantly related to good health (Eriksson and Lindström, 2006).

Although SOC was originally developed as a concept related to individuals, Antonovsky (1979, 1987b) mentioned that it could also be applicable to groups and adopted the concept of SOC to a family level (Antonovsky and Sourani, 1988). FSOC can be defined as the cognitive map of a family and orientations that enable the family to deal with stress and challenges during their lifetime. Furthermore, FSOC is the degree to which a family perceives family life as comprehensible, manageable, and meaningful (Antonovsky and Sourani, 1988). *Comprehensibility* (C) is the tendency to see the world as ordered, predictable, and explainable, which facilitates family cognitive clarification about the nature of the problems or stressors. *Manageability* (MA) is the tendency to expect the challenges generated by stressors to be manageable, which leads the family to seek the appropriate and potentially available resources. *Meaningfulness* (ME) is the tendency to see life as meaningful and important, which promotes the motivational drive to confront and deal with stressors (Antonovsky and Sourani, 1988; Ji et al., 2010).

Family sense of coherence is a strong concept to promote the health of families (Antonovsky, 1993; Hansson and Cederblad, 2004). Families, when dealing with adversity or crisis, can do it better with an enhanced *SOC*, since it involves efforts to understand the nature of the problems and act accordingly. Subjective evaluation of family members of their situation influences their coping response and adaptation (Walsh, 2012). Practitioners can help families to promote coherence by supporting the understanding of their situation and to realistically evaluate their options and actively plan their coping strategies (Hansson and Cederblad, 2004).

According to the family stress and coping literature, FSOC corresponds to the ability of a family to cope with daily difficulties and to tolerate and rebound from stressful life situations (Ji et al., 2010). Patterson (2002) considered FSOC as an important resource for the wellbeing of families as it can promote family resilience in the face of adversity. For Walsh (2016), an enhanced FSOC predicts adaption and ability to cope properly with adverse situations, higher satisfaction within the family, and greater community integration. Furthermore, MacPhee et al. (2015) referred that FSOC is associated to support the acquisition, coping strategies, adaptation to stress, and individual wellbeing. It can be seen as a family resistance resource against adversities, and it may promote the quality of life of families (Antonovsky and Sourani, 1988; Wickens and Greeff, 2005). Moreover, Eriksson and Lindström (2007) performed a systematic review with 458 studies and concluded that SOC was a determinant factor for the positive health-related quality of life of an individual.

A vast literature corroborates the idea that life events, stressors, or psychological risk factors can induce physical and/or emotional pathology (Selye, 1976; Kanner et al., 1981). Since it is not possible to live without stressors, examining possible moderating variables between the negative effects of stressors and physical and psychological health is extremely important to promote family wellbeing. FSOC could be one moderating variable since it can help families buffer the effects of and even prosper through adversities. FSOC allows family members to select the most appropriate coping behaviors to face the disruptive event or crisis, thus promoting the health of an individual (Antonovsky and Sourani, 1988).

Antonovsky and Sourani (1988) have developed FSOC Scale based on the SOC scale, thus performing considerable modifications between the two scales. The items that could not be reframed to family context were eliminated, and the items that referred to daily life issues were introduced. The inherent frame for all items was the extent to which individuals perceived family life as comprehensible, manageable, or meaningful. The sample was composed of 60 Israeli families in which all the male members of the couple were physically disabled from 2 to 10 years. The authors assessed FSOC reliability; however, they have not performed other psychometric evaluations.

Although the conceptual framework of FSOC remains pertinent and robust, families and their functioning have been changing over time since Antonovsky and Sourani (1988) developed and operationalized FSOC. Nevertheless, very few studies have used this instrument in its original version. Greeff et al. (2006) included the FSOC Scale in their study with single-parent families from Belgium but have not performed a translation and adaptation of the FSOC original scale. Ji et al. (2010) used the FSOC Scale with a United States sample of adoptive families; however, the authors did not mention an adaptation of the FSOC Scale to their sample. Speirs et al. (2016) conducted a study with a United States sample of low-income mothers and their preschool children using the FSOC Scale; however, they only explored the concurrent validity of the FSOC Scale and did not perform other psychometric evaluations of the FSOC Scale to their sample. A recent study, developed by Carneiro et al. (2019), was conducted to translate and validate the FSOC Scale in a Portuguese sample; however, the original FSOC dimensions were not preserved and therefore are not possible to guarantee that the scales are still measuring the same construct than the original one.

Other studies from Turkey, China, and Sweden (Söderhamn and Holmgren, 2004; Çeçen, 2007; Ngai and Ngu, 2011) used a shortened version of the FSOC that was translated and adapted from the version of Sagy (1998). Sagy (1998) has conducted a study with a short version of the FSOC Scale that presents a different methodological solution since it combines items from both SOC and FSOC Scales. As mentioned by the author, this FSOC short version scale “represents the extent to which the respondent sees his or her family worldview as coherent. It was measured by a self-report questionnaire, developed on the basis of the individual SOC scale, asking about the family worldview in the eyes of the adolescent” (Sagy, 1998, p. 317). This option is different from creating a shortened version directly from FSOC original scale, and those differences should be further evaluated in order to understand which could be the most suitable version of the FSOC Scale from the perspective of psychometrics. FSOC is a complex concept, and its operationalization could have different possible paths that need to be further investigated. This study decides to explore and adapt the original FSOC Scale and not the shortened version in order to follow the original FSOC Scale proposed by Antonovsky and Sourani (1988). At present, and as far as we know, the Norwegian FSOC Scale version (N-FSOC; Moen and Hall-Lord, 2016) was adapted from the original FSOC proposed by Antonovsky and Sourani (1988). The results of the Norwegian study will be further presented and debated in the “Discussion” section.

Having into account that (1) the important practical and theoretical contributions of FSOC concept to research and intervention of families, (2) there is no other instrument measuring FSOC concept, (3) the gap in research evaluating psychometric characteristics of the original FSOC, (4) the absence of a version that preserves the original framework proposed by Antonovsky and Sourani (1988), this study intends to evaluate the validity and reliability of the FSOC Scale in a sample of Portuguese families with children aged between 10 and 15 years.

## Materials and Methods

### Participants

This study was part of a larger national study about family functioning and psychosocial adjustment of children conducted with Portuguese families. This community sample comprised a total of 329 caregivers (i.e., 230 mothers, 90 fathers, 2 stepfathers, 2 stepmothers, 2 grandmothers, 1 grandparent, and 1 godmother). The only inclusion criterion was that caregivers had a child aged between 10 and 15 years. Ages of caregivers ranged from 25 to 70 years (*M* = 44.25; SD = 5.15). The mean age of children was 12.17 years (SD = 1.70). Most caregivers were married (77.2%), held a college degree (45.6%), were full-time employed (84.5%), and lived in an urban area/big city (50.5%). Full sample characteristics are reported in Table 1.

**TABLE 1:** Characteristics of the study sample (*n* = 329).

	Frequency (%)
**Relationship status**	
Married	254 (77.2)
In a relationship	8 (2.4)
Single	17 (5.2)
Divorced	43 (13.1)
Widowed	5 (1.5)
Missing values	2 (0.6)
**Educational level**	
High school or less	93 (28.3)
At least a college degree	234 (71.1)
Missing values	2 (0.6)
**Partner’s educational level**	
No partner	45 (13.7)
High school or less	80 (24.3)
At least a college degree	196 (59.6)
Missing values	8 (2.4)
**Professional status**	
Full-time	278 (84.5)
Part-time	10 (3)
Unemployment	26 (7.9)
Retirement	3 (0.9)
Independent worker	5 (1.5)
Missing values	7 (2.2)
**Partner’s professional status**	
No partner	45 (13.7)
Full-time	249 (75.7)
Part-time	10 (3)
Unemployment	11 (3.4)
Retirement	2 (0.6)
University student	3 (0.9)
Missing values	9 (2.7)
**Family household**	
1	5 (1.5)
2	27 (8.2)
3	93 (28.3)
4	137 (41.6)
5	38 (11.6)
6	9 (2.7)
7	4 (1.2)
Missing values	16 (4.9)
**Residential area**	
Urban/big city	166 (50.5)
Urban/suburbs of a big city	110 (33.4)
Semi-urban/small city	23 (7)
Rural	15 (4.6)
Village	12 (3.6)
Missing values	3 (0.9)

### Measures

Caregivers were asked to answer a brief sociodemographic questionnaire, which included age, relationship to the child, marital status, educational level, professional status, educational level of the partner, professional status of the partner, residential area, household composition, and age of the child. Caregivers were also asked to complete the FSOC Scale.

#### Family Sense of Coherence

The FSOC Scale (Antonovsky and Sourani, 1988) evaluates the global cognitive orientation of the family to see the world as comprehensible, manageable, and meaningful. The FSOC Scale was derived from the Sense of Coherence Scale (Antonovsky, 1987b) and adapted to family life. The FSOC Scale consists of 26 items scored in 7-point Likert scale with extreme anchor phrases [e.g., item 2: When you have to get things done which depend on cooperation among all members of the family, your feeling is: (1) there is almost no chance that the things will get done … (7) the things will always get done; item 3. Do you have the feeling that it is always possible, in your family, to get help one from another when a problem arises? (1) you can always get help from all family members … (7) you cannot get help from family members]. High scores correspond to a strong FSOC. The scale is composed of three subscales, namely, *ME*, MA, and C. ME refers to a motivational drive, and it corresponds to the degree to which family perceives the stressful pressures that are worthy of investment (e.g., “Family life seems to you full of interest”) and includes items 6, 8, 12, 13, 17, 19, 23, 25, and 26. MA refers to a family “scanning” or evaluation about the available resources to meet the demands posed by the stressors (e.g., “When you think of possible difficulties in important areas of family life, is the feeling, there are problems which have no solution?”) and includes items 2, 3, 5, 9, 10, 11, 16, 20, and 22. C is the cognitive orientation of family to family life taking into account the degree of predictability and explicability of family life (e.g., “Do you sometimes feel that there is no clear and sure knowledge of what’s going to happen in the family?”) and includes items 1, 4, 7, 14, 15, 18, 21, and 24. According to Antonovsky and Sourani (1988), Cronbach’s alpha for the total scale was 0.92, and 0.77, 0.80, and 0.85 for the subscales C, MA, and *ME*, respectively.

### Procedures

The initial phase of this study involved the translation and back translation of the items and instructions of the FSOC Scale. A group of five experts (i.e., psychology researchers, fluent in both Portuguese and English) have performed the translation, and after some discussion meetings, a consensual and final version was created. Other experts performed the back-translation, and other experts reviewed the translations of items and verified if the content assessed the original construct. The two final versions (i.e., translated and back translated) were compared by the authors of this study, and the necessary adjustments were performed in order to achieve the final translated version of the FSOC Portuguese Scale.

The majority of the participants have been recruited through a non-probabilistic convenience sample recruitment in private schools, learning centers, and football learning clubs from the Lisbon metropolitan area and from Setubal district/area. These participants completed the questionnaires *via* paper and pencil (P&P) or online since some parents preferred the online version. The same procedures and contacts have been established posteriorly, in order to increase the sample size, and the participants have completed the online questionnaires. The sample recruitment occurred between November 2018 and January 2021. According to the Declaration of Helsinki, all participants were given the option to clarify any question related to the content and procedures of this study. All participants sign the consent form before their participation. The study has been approved by the ISPA-University Institute’s Ethics Committee.

This study is part of a larger national study with children, their caregivers, and their teachers.

### Data Analysis

Descriptive statistics were calculated for all items of the FSOC Scale using SPSS (version 25, SPSS Inc., Chicago, IL, United States). The sensitivity of items was evaluated through Skewness (Sk) and Kurtosis (Ku) analysis. Absolute values of |Sk| and |Ku| higher than three and seven, respectively, were considered a severe violation of the normality assumption (Marôco, 2014). Structural equation models were developed using AMOS (version 18, SPSS Inc., Chicago, IL, United States).

To examine the factorial validity of the original FSOC Scale, a confirmatory factor analysis (CFA) was performed. The assumptions that determined whether these data are suitable for factor analysis were met. The sample was constituted of 329 participants, which corresponds to a ratio of at least 5–10 cases/subjects for each item/variable (Kass and Tinsley, 1979). According to Marôco (2014) and Byrne (2016), the model can be considered as having a sufferable fit when χ^2^/df (ratio of chi-square and degrees of freedom) is between 2 and 3, comparative fit index (CFI), goodness of fit index (GFI) and normed fit index (NFI) values are higher than 0.90, parsimony GFI (PGFI), and parsimony CFI (PCFI) values are higher than 0.6, root mean square error of approximation (RMSEA) and standardized root mean square residual (standardized RMR) values are lower than 0.08 and Akaike information criteria (AIC) and expected cross-validation index (ECVI) values are the lower in comparison to the other models tested. The model adjustment was performed step-by-step, according to the guidelines of Byrne (2016). Based on the statistical significance of parameter estimates, only items with a probability level of 0.05 were considered. For the loading factors, we decided to maintain items with standardized regression weights equal to or above 0.40 and squared multiple correlations equal to or above 0.15.

To assess convergent-related validity, the average variance extracted (AVE) was estimated. Values of AVE above 0.50 were considered indicative of the convergent-related validity of constructs (Marôco, 2014). Discriminant-related validity was explored through the comparison of the squared correlation of inter-factors with the AVE of each individual factor. Evidence of discriminant-related validity can be found when the squared correlation between factors is smaller than the individual AVE (Marôco, 2014). Reliability was investigated through internal consistency estimates, namely, the composite reliability (CR) and the standard Cronbach’s alpha coefficient (α) for the FSOC total scale and its dimensions (Marôco, 2014). Cronbach’s alpha values ≥0.70 and CR values ≥0.80 are considered acceptable/adequate.

## Results

The results presentation followed the Standards for Educational and Psychological Testing framework.

### Descriptive Statistics

Items 1, 3, 5, 6, 9, 10, 13, 15, 18, 21, 22, 24, 25, and 26 were scored in reverse, as designated by Antonovsky and Sourani (1988). All missing values (*n* = 22) were replaced by the mean. The average FSOC Scale was 74.01 (SD = 25.07). As shown in Table 2, FSOC Scale items’ descriptive statistics indicated that the entire seven-point Likert scale was used for all items, with answers ranging from one to seven. Distribution of item presented acceptable Sk (−0.062 < Sk < 2.325) and Ku (−1.307 < Ku < 7.103) values (Marôco, 2014; Kline, 2016). Having into account that only item 17 has a Ku > 7 (Ku = 7.103), we decided at this phase to maintain this item.

**TABLE 2:** Descriptive and distributional properties of items of family sense of coherence (FSOC).

Item	Mean	SD	Min	Max	Skewness	Kurtosis
FSOC1	3.170	1.905	1	7	0.691	−0.735
FSOC2	2.552	1.519	1	7	1.124	0.700
FSOC3	3.353	2.267	1	7	0.506	−1.307
FSOC4	2.984	1.743	1	7	0.587	−0.739
FSOC5	2.841	2.258	1	7	0.913	−0.774
FSOC6	3.378	1.816	1	7	0.362	−0.880
FSOC7	3.331	1.650	1	7	0.295	−0.830
FSOC8	2.757	1.653	1	7	1.040	0.480
FSOC9	3.973	1.415	1	7	−0.062	−0.318
FSOC10	3.131	2.704	1	7	0.704	−0.847
FSOC11	2.736	1.314	1	7	0.851	0.905
FSOC12	3.158	1.202	1	7	1.277	1.643
FSOC13	2.796	2.223	1	7	0.993	−0.591
FSOC14	2.474	1.403	1	7	0.948	0.390
FSOC15	3.663	1.906	1	7	0.250	−1.153
FSOC16	1.884	1.062	1	7	1.839	4.787
FSOC17	1.638	0.997	1	7	2.325	7.103
FSOC18	3.262	1.950	1	7	0.630	−0.873
FSOC19	1.920	1.118	1	7	1.679	3.846
FSOC20	2.143	1.182	1	7	1.352	2.041
FSOC21	3.168	1.831	1	7	0.625	−0.723
FSOC22	3.345	1.706	1	7	0.618	−452
FSOC23	2.146	1.576	1	7	1.671	2.113
FSOC24	2.687	2.139	1	7	0.403	−1.079
FSOC25	6.05	1.46	1	7	1.076	−0.350
FSOC26	3.126	1.768	1	7	0.730	−0.552

### Confirmatory Factor Analysis, Convergent-Related Validity, and Discriminant-Related Validity and Reliability

The CFA was performed to determine the model goodness of fit with the variables and structure proposed by Antonovsky and Sourani (1988). The variables used were the variables for FSOC which consisted of three constructs named ME, MA, and C (C). Three analyses were conducted.

#### Model 1

The first CFA test was conducted with the original scale characteristics. This model did not present an acceptable fit to the data [χ^2^ (296) = 1,487.503, *p* < 0.001; χ^2^/df = 5.03; CFI = 0.759; GFI = 0.63; PGFI = 0.532; PCFI = 0.691; NFI = 0.718; RMSEA = 0.111; AIC = 1,597.503; ECVI = 4.870; SRMR = 0.134] with regression weight *p*-values over 0.05 and with loadings below 0.40 for items 2, 7, 8, and 11. These items were removed, and another CFA test was conducted.

#### Model 2

After adjusting the model, the model fit was still not acceptable to the data [χ^2^ (206) = 1,153.975, *p* < 0.001; χ^2^/df = 5.60; CFI = 0.798; GFI = 0.671; PGFI = 0.546; PCFI = 0.712; NFI = 0.766; RMSEA = 0.118; AIC = 1,247.975; ECVI = 3.805; SRMR = 0.132]. Items 4, 9, 12, 14, 16, 17, 19, 20, and 23 presented loadings below 0.40 and were, therefore, eliminated.

#### Model 3

**FIGURE 1:**
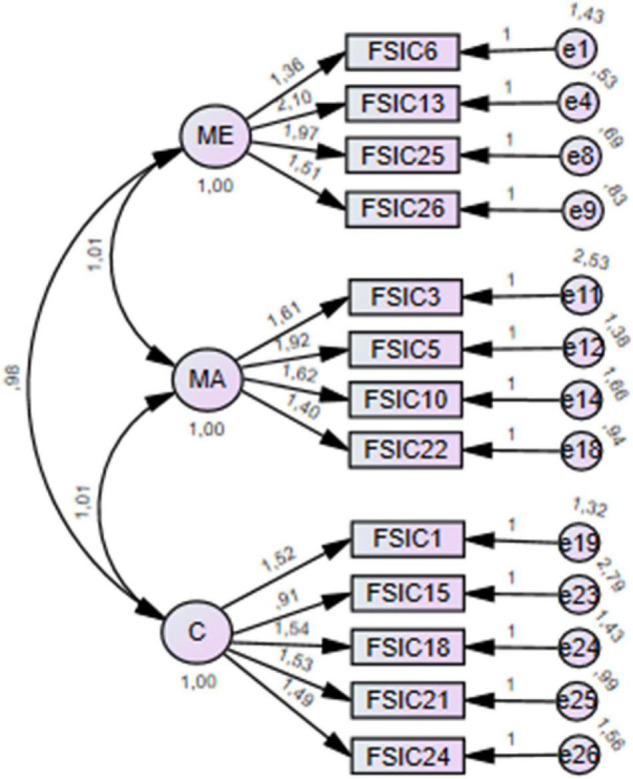
Final model for confirmatory factor analysis.

The final model (Figure 1) presented a good fit to the data [χ^2^ (62) = 139.924, *p* < 0.001; χ^2^/df = 2.26; CFI = 0.979; GFI = 0.939; PGFI = 0.639; PCFI = 0.778; NFI = 0.963; RMSEA = 0.062; AIC = 197.924; ECVI = 0.602; SRMR = 0.025]. χ^2^/df presented a sufferable fit to the data, GFI, PGFI, and PCFI presented a good fit to the data, and CFI, NFI, RMSEA, and SRMR presented a very good fit to the data. χ^2^, AIC, and ECVI were the lowest by comparison with the values reported for models 1 and 2 (χ^2^: 1,487.503 vs. 1,153.975 vs. 139.924; AIC: 1,597.503 vs. 1,247.924 vs. 197.924; ECVI: 4.870 vs. 0.603 vs. 0.602), reflecting better fit.

**FIGURE 2:**
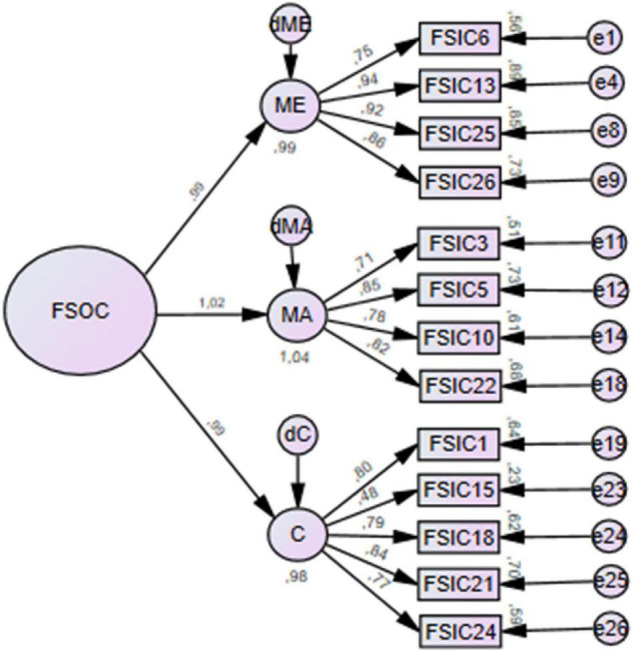
Second-order factor structure model for confirmatory factor analysis.

Although the model presented a good fit to the data, covariance values of the MA dimension presented values higher than 1. This could suggest, according to Marôco (2014), a second-order factor structure. The second-order factor was added as shown in Figure 2. The introduction of a second-order factor increased the covariance value for the MA dimension.

Convergent-related validity was assessed through the AVE. For all the dimensions, AVE was less than acceptable (AVE_*ME*_ = 0.338; AVE_*MA*_ = 0.281; AVE_*C*_ = 0.309). However, the AVE of the FSOC Scale was acceptable (AVE_*TOTAL*_ = 0.689). Systematic removal of each item had no impact on the AVE scores. Convergent-related validity can also be considered if the correlations between the items of each dimension are considered strong and positive. Table 3 shows Pearson’s correlation values. According to Cohen (1988), Pearson’s correlation values can be considered: weak from 0.10 to 0.29, moderate from 0.30 to 0.49, and strong when the values are higher than 0.50. Only the correlations with item 15 are considered moderate, and all the others are strong correlations, indicating convergent-related validity.

**TABLE 3:**
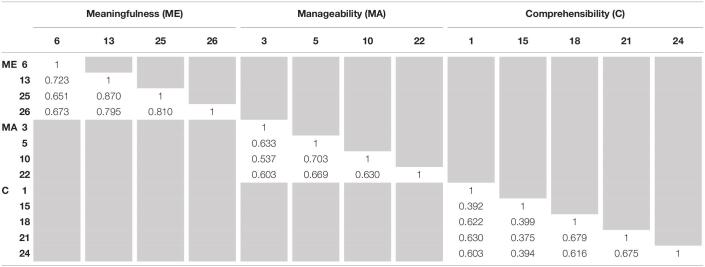
Pearson’s R correlation between FSOC Scale items.

No evidence of discriminant-related validity was found for any dimension given that the inter-factor squared correlations were higher than scores of their individual AVE (Table 4). Systematic removal of each item had no influence on covariances values. Similarly, the addition of a second-order factor as not influenced the covariances values as presented before.

**TABLE 4:** Discriminant-related validity of the FSOC Scale.

Dimensions	Squared correlations	Discriminant-related validity
Meaningfulness – Manageability	1.026	No
Meaningfulness – Comprehensibility	0.968	No
Manageability – Comprehensibility	1.016	No

The internal consistencies and CR for the FSOC Scale and respective dimensions are listed in Table 5. FSOC Scale confirms excellent reliability (α = 0.956; CR = 0.974), while the three dimensions confirm good reliability (0.853 < α < 0.923, and 0.608 < CR < 0.685), with excellent values of internal consistency (α_*ME*_ = 0.923; α_*MA*_ = 0.866; α_*C*_ = 0.853) and marginal CR values (CR_*ME*_ = 0.669; CR_*MA*_ = 0.608; CR_*C*_ = 0.685). Systematic removal of each item had no impact on CR scores. For example, removing item 15 which has the lower standardized regression weight (λ = 0.48) decreased CR value, as well as AVE, and also fit indexes. Nevertheless, FSOC Scale presented excellent values of internal consistency and CR.

**TABLE 5:** Average variance extracted and reliability analysis for FSOC Scale and FSOC Scale dimensions.

	Meaningfulness	Manageability	Comprehensibility	Family Sense of Coherence Scale
Cronbach’s Alpha	0.923	0.866	0.853	0.956
CR	0.669	0.608	0.685	0.974
AVE	0.338	0.281	0.309	0.689

## Discussion

The FSOC Scale (Antonovsky and Sourani, 1988) was created and developed over 30 years ago from a strong and pertinent conceptual framework regarding families and their salutogenic functioning, and to the best of our knowledge, there is no other instrument with the same purpose. The FSOC Scale is a highly context-dependent measure that could vary according to the different cultural contexts and different samples, which highlights the importance to validate and adapt the FSOC Scale to different countries and different family samples. This study aimed to explore the factorial structure, validity, internal consistency, and composite reliability of the FSOC Scale, preserving the original structure and dimensions. The aim of this study was in line with the systematic review of Eriksson and Lindström (2005) about validity studies of SOC Scale in which they recommend that “(…) there is no need to develop new SOC versions. There is rather a need of consolidation and a standardization of the instruments” (Eriksson and Lindström, 2005, p. 463).

The findings of this study supported the three-factor structure proposed by the authors of the original FSOC Scale (Antonovsky and Sourani, 1988), however, with only 13 items. For some factors, their regression weights were low or had non-significant saturation levels, resulting in the elimination of 13 items. The second-order factor model showed an equally good fit to the data but did not solve the increased values of the covariances. A possible explanation for this is that there are other possible variables promoting the variability of dimension. Nevertheless, the existence of a second-order factor confirms that the FSOC is a latent variable, which is reflected in the three dimensions, and the dimensions are reflected by the items that compose them (Marôco, 2014). FSOC Scale was developed after the SOC Scale and was described as an adaptation of the SOC scale for the family context (Antonovsky and Sourani, 1988). Regarding the structure of the SOC Scale, the three-factor structure is still not completely consensual in the literature: some studies identified a five-factorial structure, others found a one-factor structure, and others proposed a second-order factorial structure (Eriksson and Lindström, 2005). Nevertheless, our findings are in accordance with the study by Eriksson and Lindström (2005) that defends that a three-factor structure and a second-order factor model could be a better fit to the data, compared to other factor structure hypotheses.

The findings revealed a good model fit and good reliability for the 13-item model composed of the same three dimensions as the original FSOC Scale. Although the original instrument had 26 items, our results showed that factor regression weights for some factors were low or had non-significant saturation levels, resulting in the elimination of 13 items. The *ME* and MA dimensions ended up with four items each, and the C dimension ended up with five items. FSOC Scale presented higher reliability values than its dimensions. It is possible that the elimination of 13 items could have resulted in the decreasing of FSOC dimension reliabilities. Nonetheless, as Antonovsky (1993) and Eriksson and Lindström (2005) postulated, this scale should be used as the measurement of the whole and not to examine the three dimensions separately. In fact, in some studies (Moen and Hall-Lord, 2016), the authors only presented total scores for reliability and validity. The FSOC Scale model proposed in this study reveals very good values of reliability and convergent-related validity.

A study conducted by Carneiro et al. (2019) proposed a Portuguese version of FSOC. Although the results showed a suitable version of FSOC, the factorial structure was modified, as some items were incorporated in different dimensions, and the final solution proposed different concepts for all three dimensions, deflecting from the original instrument and theoretical basis proposed by Antonovsky and Sourani (1988). Also, one of the dimensions proposed showed unacceptable values of validity and reliability. It is our understanding that these modifications (even though they were theoretically sustained) implied a deviation from the original concept and also from the theoretical contributions developed by Antonovsky. Although the aim of the study proposed by Carneiro et al. (2019) was different from this study, this is the only study conducted with a Portuguese sample examining psychometric characteristics of the FSOC Scale.

To the best of our knowledge, although other studies analyzed FSOC Scale psychometric properties in Turkey, China, Sweden, and Norwegian (Söderhamn and Holmgren, 2004; Çeçen, 2007; Ngai and Ngu, 2011; Moen and Hall-Lord, 2016), only the Norwegian study have used the FSOC Scale as proposed by Antonovsky and Sourani (1988), while the other studies used a short version of FSOC Scale proposed by Sagy (1998) with a 12-item model. FSOC short version of Sagy (1998) is the combination of SOC Scale items adapted to family context (e.g., “To what extent do you see a clear future for your family and how do you expect to see your family in 3 years?” or “Do you have the feeling that you are being treated unfairly by your family?”) with FSOC Scale items (e.g., When you have to get things done which depend on cooperation among all members of the family, your feeling is …). The author has neither explained his decision to compose the FSOC Scale short version with items from both SOC and FSOC Scale nor the reasons for choosing those particular 12 items. Study by Sagy (1998) presented adequate values of FSOC Scale reliability (α = 0.81); however, no other psychometric evaluations were performed. Since the FSOC Scale short version proposed by Sagy (1998) is not fully sustained methodologically or psychometrically, we have chosen to perform our analysis with the FSOC original scale proposed by Antonovsky and Sourani (1988).

Moreover, the abovementioned FSOC short versions used in Turkey, China, Sweden, and Norwegian (Söderhamn and Holmgren, 2004; Çeçen, 2007; Ngai and Ngu, 2011; Moen and Hall-Lord, 2016) presented the same items, however, with different semantic formulations without mentioning the reasons for those modifications. For example, in the FSOC Chinese version (Ngai and Ngu, 2011), item 12 was presented as “has a family member you trusted ever disappointed you?” and the same item in the FSOC Swedish version (Söderhamn and Holmgren, 2004) was presented as “has it ever happened that people in your family on whom you counted on disappointed you?,” also the item 8 was presented as “Do you feel that your family treats you fairly?” and as “Do you have the feeling that you are being treated unfairly by your family?” by the FSOC Chinese version and FSOC Swedish version, respectively. Although the Norwegian version (N-FSOC) (Moen and Hall-Lord, 2016) was not published in an indexed journal, we decided to report their findings because it is, as far as we know, the only version developed from the original FSOC Scale. In the N-FSOC study, only construct-related validity and reliability were assessed. Internal consistency was assessed by Cronbach’s alpha that showed an adequate value (α ≥ 0.87) for the three groups in this study. The construct-related validity was assessed by Pearson’s correlation with other instruments (e.g., SOC). Since no factor analysis (confirmatory or exploratory) was performed, it is not possible to compare the N-FSOC factorial structure with the FSOC factorial structure of this study.

There are some study limitations that should be taken into account when interpreting the results. This study uses a cross-sectional design and did not evaluate test-retest stability or predictive validity of the FSOC. Future studies are necessary to assess the stability of this measure over time. Some participants in this study answered the questionnaires during the lockdowns imposed by governments due to the coronavirus disease 2019 (COVID-19) pandemic, which could influence routines, processes, and life perspectives of families.

Another study limitation is the convenience sampling method since it does not enable the determination of the representativeness of the study sample. Further research is needed to help establish the generalization of findings. FSOC was developed many years ago and has not been explored and evaluated in terms of its factorial structure; therefore, it is very important that future studies could evaluate this new proposal as well as to test this scale with different samples. Finding normative values or cutoff points of FSOC will be important for clinical use.

The sample of this study was a community sample of Portuguese caregivers while the original sample of FSOC was composed of Israeli families from the National Security Institute. Not only the cultural context of the two studies is very different but also the families from both studies had different life situations since the Israeli families have at least one member with a disability and the Portuguese families did not. It is possible that the number of FSOC reverse items is related to the emotional state or affective disposition of these families who experience a challenging situation in their context by having one of the members with a physical disability. It is also possible that the cultural context may cause differences in the psychometric properties and adequacy of the instrument. Thus, future studies should qualitatively adapt the scale and clarify possible cultural differences.

The presented findings comprise an important contribution to this field and propose a strong instrument to evaluate FSOC and to indicate new paths to improve it, standing along with the contributions of Antonovsky and Sourani (1988). To the best of our knowledge, this is the first study exploring the factorial structure, validity, and reliability of the FSOC Scale, as Antonovsky and Sourani (1988) proposed. The exponential interest in family systems and processes referred by researchers and family practitioners (Gomes et al., 2019) combined with the lack of suitable Portuguese measures to assess family resilience and coping strategies and specifically FSOC makes FSOC Scale extremely useful and relevant in research and practice with families since this is the only instrument developed to evaluate FSOC. This study returns to a family concept that has been overlooked in the last decades. Although there are several studies about how families adjust to stressful life events, FSOC is an important concept that promotes family wellbeing since it provides the motivational, perceptual, and behavioral basis to deal with stress during their lifetime. From a transcultural perspective, this study could be an important asset to cross-cultural comparative studies that intend to evaluate similarities and differences about the family functioning in different countries or cultures. By deepening the knowledge about strategies of family and ways to deal with adversity, family therapists could upgrade their therapeutic tools to help families deal with difficulties in healthier ways.

## Data Availability Statement

The raw data supporting the conclusions of this article will be made available by the authors, without undue reservation.

## Ethics Statement

The studies involving human participants were reviewed and approved by the ISPA’s Ethics Committee. The patients/participants provided their written informed consent to participate in this study.

## Author Contributions

FC recruited the sample. FC and VS performed all the statistical analyses and wrote all article sections. PC contributed to the study design, analysis plan, and reviewed the article. IL contributed to the study design and reviewed the article. All authors reviewed the manuscript and contributed to it in a meaningful way.

## Conflict of Interest

The authors declare that the research was conducted in the absence of any commercial or financial relationships that could be construed as a potential conflict of interest.

## Publisher’s Note

All claims expressed in this article are solely those of the authors and do not necessarily represent those of their affiliated organizations, or those of the publisher, the editors and the reviewers. Any product that may be evaluated in this article, or claim that may be made by its manufacturer, is not guaranteed or endorsed by the publisher.
